# Supplementary material for: Multi-Omics Identifies Circulating miRNA and Protein Biomarkers for Facioscapulohumeral Dystrophy
Source: J Pers Med. 2020 Nov 19;10(4):236. doi: 10.3390/jpm10040236 (PMC7711540; doi:10.3390/jpm10040236)
Supplement: Supplementary file 1 [file jpm-10-00236-s001.zip › Table S2_Total proteomic changes in FSHD plasma as detected via LC-MS MS.docx]

**Table S2.** Circulating proteins identified as dysregulated in FSHD plasma via LC-MS/MS, based on signal intensity

| **UniProt ID** | **Protein name** | **Gene Name** | **Total Unique peptides N (Control/FSHD)** | **↑**  **or**  **↓** | **p-value** | **Known roles in muscle / disease pathways** |
| --- | --- | --- | --- | --- | --- | --- |
| Q15848 | Adiponectin | ADIPOQ | 12(5/7) | ↑ | 0.0142 | increased in DMD; adipokine that regulates metabolism in muscle |
| P04114 | Apolipoprotein B-100 | APOB | 39(14/25) | ↓ | 0.0167 | lipid transport, elevated in heart disease |
| P55056 | Apolipoprotein C-IV | APOC4 | 20(9/11) | ↓ | 0.0139 | lipid transport from intestine to muscle |
| P02655 | Apolipoprotein C-II | APOC2 | 33(13/20) | ↓ | 0.0058 | lipid transport; genetic marker for myotonic dystrophy |
| P23528 | Cofilin-1 | CFL1 | 18(8/10) | ↑ | 0.0037 | actin filament organization and depolymerization |
| P02741 | C-reactive protein | CRP | 11(3/8) | ↑ | 0.0328 | elevated in myositis; elevated / biomarker for IBD |
| P01034 | Cystatin-C | CST3 | 23(10/13) | ↓ | 0.0283 | biomarker for cardiovascular and kidney diseases |
| Q12805 | Fibulin-3 | EFEMP1 | 13(5/8) | ↑ | 0.0013 | plasma biomarker for mesothelioma; retinal dystrophy |
| P03951 | Coagulation factor XI | F11 | 30(12/18) | ↓ | 0.0381 | Noonan syndrome & hypotonia; near *D4Z4* genomic locus; coagulation factor |
| P00488 | Coagulation factor XIII A chain | F13A1 | 13(5/8) | ↑ | 0.0227 | hypertension, angiotensin II, coagulation |
| P23142 | Fibulin-1 | FBLN1 | 23(9/14) | ↑ | 0.0037 | positive regulation of fibroblast proliferation |
| P00738 | Haptoglobin | HP | 39(14/25) | ↑ | 0.0485 | up in DMD plasma; associated with IBD, arthritis and other inflammatory diseases |
| P05019 | Insulin-like growth factor I | IGF1 | 26(11/15) | ↑ | 0.0398 | hypertrophy, development, satellite cells, regeneration |
| P01857 | Ig gamma-1 chain C region | IGHG1 | 27(10/17) | ↓ | 0.0262 | down in endothelial corneal dystrophy |
| P03952 | Plasma kallikrein | KLKB1 | 38(14/240 | ↓ | 0.0408 | inflammation and coagulation; near *D4Z4* |
| P07737 | Profilin-1 | PFN1 | 19(7/12) | ↑ | 0.0003 | actin cytoskeleton organization |
| Q92954 | Proteoglycan 4; | PRG4 | 23(8/15) | ↓ | 0.0377 | TLR4; anti-inflammatory, down in arthritis |
| P04070 | Vitamin K-dependent protein C | PROC | 22(9/13) | ↓ | 0.0376 | anti-inflammatory, down in chronic inflammatory diseases such as IBD |
| P41222 | Prostaglandin-H2 D-isomerase | PTGDS | 15(7/8) | ↑ | 0.0463 | neuromodulator; smooth muscle contraction |
| P61224 | Ras-related protein Rap-1b, 1a | RAP1B | 15(6/9) | ↑ | 0.0111 | GTP-binding protein |
| P05109 | Protein S100-A8 | S100A8 | 18(5/13) | ↑ | 0.0042 | TLR4; pro-inflammation, up in rheumatic diseases and IBD |
| P0DJI9 | Serum amyloid A-2 protein | SAA2 | 5(1/4) | ↑ | 0.0339 | IBD, Induce Pathogenic Th17 Cells |
| Q13103 | Secreted phosphoprotein 24 | SPP2 | 13(7/6) | ↑ | 0.0431 | pro-inflammatory, NF-κB; blood pressure; bone health |
| P37802 | Transgelin-2 | TAGLN2 | 15(7/8) | ↑ | 0.0416 | marker of differentiated smooth muscle |
| P60174 | Triosephosphate isomerase | TPI1 | 8(2/6) | ↑ | 0.0479 | glycolysis |
| P67936 | Tropomyosin alpha-4 chain | TPM4 | 17(8/9) | ↑ | 0.0421 | actin organization, muscle contraction |
| Q6EMK4 | Vasorin | VASN | 23(9/14) | ↓ | 0.0481 | binds TGF-β; vascular smooth muscle |
| P15924 | Desmoplakin | DSP | 16(7/9) | ↓ | 0.020 | down in *mdx* muscle; intercellular junctions; cardiomyopathy |
| P29401 | Transketolase | TKT | 6(2/4) | ↑ | 0.030 | connects pentose phosphate pathway to glycolysis |
| F5H7V9 | Tenascin | TNC | 4(1/3) | ↓ | 0.041 | extracellular matrix, adhesion modulation |
| O95497 | Pantetheinase | VNN1 | 10(4/6) | ↑ | 0.008 | upregulated in IBD |
| P08779 | Keratin, type I cytoskeletal 16 | KRT16 | 13(7/6) | ↑ | 0.009 | elevated with S100A8 in skin disorders, psoriasis |

DMD = Duchenne muscular dystrophy, IBD = inflammatory bowel disease, mdx = mouse model for Duchenne muscular dystrophy X-linked.
